# CSPG4: a prototype oncoantigen for translational immunotherapy studies

**DOI:** 10.1186/s12967-017-1250-4

**Published:** 2017-07-01

**Authors:** Valeria Rolih, Giuseppina Barutello, Selina Iussich, Raffaella De Maria, Elena Quaglino, Paolo Buracco, Federica Cavallo, Federica Riccardo

**Affiliations:** 1 Department of Molecular Biotechnology and Health Sciences, Molecular Biotechnology Center, University of Torino, 10126 Turin, Italy; 2 Department of Veterinary Sciences, University of Torino, 10095 Grugliasco, Italy

**Keywords:** Chondroitin sulfate proteoglycan-4 (CSPG4), Cancer immunotherapy, Comparative oncology, DNA vaccination

## Abstract

Thanks to striking progress in both the understanding of anti-tumor immune response and the characterization of several tumor associated antigens (TAA), a more rational design and more sophisticated strategies for anti-tumor vaccination have been possible. However, the effectiveness of cancer vaccines in clinical trial is still partial, indicating that additional studies are needed to optimize their design and their pre-clinical testing. Indeed, anti-tumor vaccination success relies on the choice of the best TAA to be targeted and on the translational power of the pre-clinical model used to assess its efficacy. The chondroitin sulfate proteoglycan-4 (CSPG4) is a cell surface proteoglycan overexpressed in a huge range of human and canine neoplastic lesions by tumor cells, tumor microenvironment and cancer initiating cells. CSPG4 plays a central role in the oncogenic pathways required for malignant progression and metastatization. Thanks to these features and to its poor expression in adult healthy tissues, CSPG4 represents an ideal oncoantigen and thus an attractive target for anti-tumor immunotherapy. In this review we explore the potential of CSPG4 immune-targeting. Moreover, since it has been clearly demonstrated that spontaneous canine tumors mimic the progression of human malignancies better than any other pre-clinical model available so far, we reported also our results indicating that CSPG4 DNA vaccination is safe and effective in significantly increasing the survival of canine melanoma patients. Therefore, anti-CSPG4 vaccination strategy could have a substantial impact for the treatment of the wider population of spontaneous CSPG4-positive tumor affected dogs with a priceless translational value and a revolutionary implication for human oncological patients.

## Background

It is when oncology meets immunology that cancer immunotherapy begins. Strengthen the patient’s own immune response against cancer cells represents one of the most challenging and exciting concept of active cancer immunotherapy [1].

Neoplastic initiation and progression are accompanied by the accumulation of several genetic modifications in somatic cells. The transcriptional and mutational landscape of tumors indicates that there is a clear opportunity for the immune system to distinguish tumor cells from healthy tissue and trigger a specific attack against both conserved and mutated tumor-associated antigens (TAA) [2, 3]. Indeed, a high number of potential TAA has been identified for each individual type of cancer [2, 4], making a more rational design and more sophisticated strategies for targeted anti-tumor vaccination a reality. In this evolving scenario, DNA vaccines represent an attractive and potentially effective tool for antigen-specific immunotherapy. Theoretically any mutated, abnormally expressed or over-expressed TAA could be exploited as a target to design a specific anti-cancer DNA vaccine, however, despite extensive effort by academia and industry, only one cancer vaccine has been approved by the U.S. Food and Drug Administration (FDA) so far [5]. The obstacles in successfully translating the virtually infinite number of potentially targetable TAA into effective anti-cancer vaccines have highlighted that being a TAA does not necessarily mean to be a good target for immunotherapy. Moreover, despite the existence of several successful immunotherapeutic strategies in mouse cancer models, their translation to human malignances fails because of unacceptable toxicity or a lack of efficacy [6, 7].

For these reasons, the clear need for more refined and predictive pre-clinical models becomes apparent. Therefore, in 2003 the National Cancer Institute’s Center for Cancer Research introduced the Comparative Oncology Program in order to foster the study of naturally occurring cancer in pet animals as models of human tumors [8, 9]. A European initiative with a similar purpose—the LUPA project—has also recently been launched [10]. The aim of comparative oncology is to integrate the naturally occurring cancers seen in veterinary patients into a more general study of cancer biology and therapy, to benefit both veterinary and human oncological patients [8, 9].

This review will highlight the role of chondroitin sulfate proteoglycan-4 (CSPG4) as one of the most attracting TAA identified so far for translational immunotherapy studies in both human and veterinary field.

## The oncoantigen concept

Displaying a low level of expression in healthy tissues and a high level of expression in tumors, in addition to own a “driving” role in the promotion of cancer development—pushing the progression of a neoplastic lesion from one stage to the next—are the features of “ideal” immunotherapeutic targets.

The term “oncoantigen” was coined to describe those TAA that display the above-mentioned characteristics [11, 12]. Their stable expression throughout the various tumor developmental stages and the key role in tumor growth and survival make oncoantigens normally not susceptible to immunoediting and even if oncoantigen-loss variants might occur, they would have a crippled tumorigenic potential and undergo negative selection [13]. According to their localization, oncoantigens can be divided in three classes: (a) those expressed on the cell surface (Class I oncoantigens; receptors, adhesion molecules, etc.); (b) those present in the tumor microenvironment (Class II oncoantigens; growth factors, angiogenic factors, etc.); and (c) those that are intracellular proteins (Class III oncoantigens; non-receptor tyrosine kinases, transcription factors, cell cycle molecules). Being susceptible to the attack of both T cells and antibodies, Class I oncoantigens are considered the ideal targets for effective anti-cancer immunotherapeutic strategies [12, 14, 15].

The expression level in neoplastic and/or cancer initiating cells (CIC), the cellular localization and the role in oncogenic pathways, together with the percentage of patients with tumors expressing a given TAA, are characteristics also used by the National Cancer Institute of the U.S. to prioritize cancer antigens for the development of focused immunotherapeutic vaccines [16]. The CSPG4 belongs to this list of promising TAA [16].

## CSPG4 identification card: structure, function and distribution

CSPG4, high molecular weight-melanoma associated antigen (HMW-MAA) and melanoma chondroitin sulfate proteoglycan (MCSP) are different names to label a cell surface molecule with unique structural characteristics. CSPG4 was first characterized on human melanoma cells more than 30 years ago [17]; in the same period, other studies have identified the nerve/glial antigen 2 (NG2) that is the rat orthologue of CSPG4 [17, 18]. The complete sequence of CSPG4 expressed by human melanoma cells was published in 1996 [19]: the gene, located on chromosome 15:24q2, is composed of 10 exons and the cDNA sequence length is of 8071 base pair (bp) encoding an open reading frame of 2322 amino acids (aa) [20, 21]. CSPG4 is a transmembrane protein that can be expressed on the cells either as a N-linked glycoprotein of ~250 kDa or as a ~450 kDa N-linked glycoprotein associated to a proteoglycan component [21]. The extracellular region of CSPG4 contains the sequences for the glycosylation with the chondroitin sulfate (CS) chains that can influence the distribution of the protein on the cell surface [22]. The large extracellular domain (1–2221 aa) is composed of three subdomains D1–D3 [17], encompassing binding sites for extracellular matrix (ECM) proteins, growth factors, integrins, matrix metalloproteinases and lectins. In particular, the D1 subdomain (1–640 aa) is an N-terminal globular domain consisting of two laminin G-type regions (LGR) and abundant disulfide bonds critical to maintain the tertiary structure. In these regions there are sites for ligand binding, for integrins interactions and cell matrix or cell–cell connections. The D2 subdomain (641–1590 aa) is composed of a succession of 15 “CSPG repeat” motifs, some of which are binding sites for CS chains covalently attached, collagens V and VI, or FGF and PDGF growth factors [23, 24]. The D3 subdomain (1591–2221 aa) is a membrane proximal globular domain with binding sites for the galectin-3, β1 integrins, and other lectins (e.g., p-selectin) [25, 26]. This juxtamembrane region contains also some proteolytic sites for the CSPG4 cleavage. Actually, CSPG4 fragments have been widely described [27, 28]; tryptic products have been detected in sera from healthy donors and cancer patients but their biological and clinical significance is still fairly unknown [26, 29, 30].

**Fig. 1:**
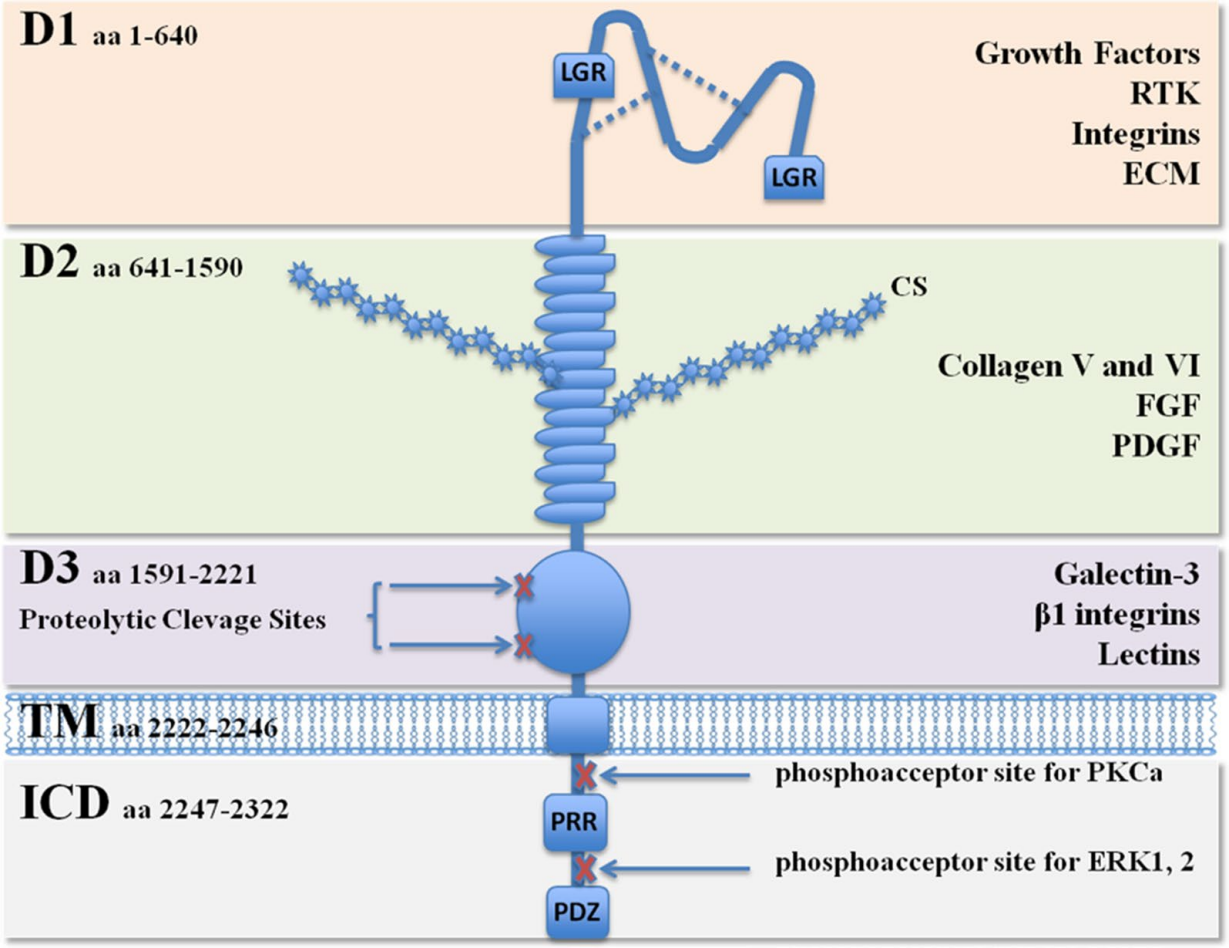
Schematic drawing of CSPG4 protein. *CS* chondroitin sulfate, *D1, D2* and *D3* subdomains of the extracellular portion, *TM* transmembrane domain, *ICD* intracellular domain, *LGR* laminin G-type regions, *PRR* proline-rich region, *PDZ* PDZ binding domain. The most important molecules interacting with each subdomain of CSPG4 are indicated on the *right*. *RTK* receptor tyrosine kinase, *ECM* extracellular matrix, *FGF* fibroblast growth factors, *PDGF* platelet-derived growth factor, *PKC* protein kinase C, *ERK* extracellular signal-regulated kinases

The extracellular portion of the CSPG4 is followed by a 25-aa transmembrane sequence (2222–2246 aa) joined to a 75-aa cytoplasmatic domain (2247–2322 aa) encompassing sites critical for CSPG4 function: (a) the PDZ binding domain on the carboxyl terminal portion that is the site for the attachment of scaffold proteins such as MUPP1, syntenin and GRIP1 [31]; (b) two multiple threonine phosphoacceptor sites phosphorylated by PKCα and extracellular signal-regulated kinase (ERK) 1 and 2 [32] and (c) a proline-rich region (PRR) that should promote other protein interactions [33]. A schematic depiction of CSPG4 structure is represented in Fig. 1.

As a membrane-spanning molecule, CSPG4 plays an important role in the communication between the outside and the inside compartments of the cell. Notably, CSPG4 is devoid of a catalytic activity [34]; however, it can participate in signal transduction starring as co-receptor. Thanks to its extended extracellular arm, CSPG4 could “capture” and present growth factors to different tyrosine kinase receptors (RTK) or link the molecules of the ECM, including collagen type II, V, and VI, laminin, tenascin and fibronectin to integrins, potentiating and sustaining the activated pathways [17, 35]. Along with enhancing growth factor activity and integrin-mediated pathways, CSPG4 could also serve as a direct cell surface receptor for ECM components [23, 36]. The intracellular domain serves as “central recruitment” for scaffolding proteins linking CSPG4 to intracellular signaling pathways and to the actin cytoskeleton [37]. All these features highlight the central key role of CSPG4 in orchestrating cell proliferation, adhesion, migration and survival.

CSPG4 is involved mainly in tissue development or homeostasis and retains a limited expression in adult healthy tissues [26]. Its expression was initially found in a limited number of normal cell types, including vascular pericytes, articular chondrocytes, and the microglia in the central nervous system. More recently, CSPG4 mRNA expression has been detected in a broader range of tissues, including brain, gastrointestinal tract and endocrine organs, where it appears to be distinctive of precursor/progenitor cells of epithelial and mesodermal origin [38–41]. However, in normal tissues CSPG4 mRNA expression levels do not correlate with the protein expression [41].

## CSPG4 as a prototype oncoantigen in human tumors

Marked by a restricted distribution in normal tissues, CSPG4 is over-expressed in several haematological and solid neoplastic conditions besides melanomas, including oligodendrocytomas, gliomas, childhood acute lymphoblastic leukemia and acute myeloid leukemia, renal cell carcinomas, chondrosarcomas, pancreatic and triple-negative breast carcinomas. In neoplastic lesions CSPG4 is highly expressed on both malignant cells and activated pericytes within the tumor microenvironment [42, 43]. The association between CSPG4 expression and patients’ poor prognosis suggests that CSPG4 is directly involved in the neoplastic progression [18, 24, 29, 41, 44–47]. The best-established implication regards the link between CSPG4 and melanoma progression that was first appreciated as a result of its widespread expression in the majority (85% or greater) of human melanomas [21, 48, 49].

As a consequence of its ability to coordinate several intracellular pathways regulating different cell functions, CSPG4 becomes involved in tumorigenesis at multiple levels [41, 46]. Specifically, the over-expression of CSPG4 could sustain a high proliferative phenotype of tumor cells. By means of its ability to act as co-receptor, binding to and presenting growth factors to their cognate RTK, CSPG4 could potentiate the activation of the mitogen-activated protein kinases (MAPK) pathway, resulting in the selective growth and survival advantage of CSPG4-positive tumor cells [50, 51]. Also in those melanomas in which the BRAF^V600E^ mutation determines a constitutive activation of the downstream MAPK pathway positively impacting on cell proliferation, the expression of CSPG4 is required to maximize the tumorigenic effect [52]. CSPG4 over-expression has also been involved in cancer cell progression through the regulation of intracellular pathways implicated in tumor cell adhesion and migration. Several studies have demonstrated that integrin-mediated signaling can be super-activated in the presence of CSPG4 over-expression, as compared to stimulation of integrins alone [52–54]. By binding ECM molecules and interacting with integrins, CSPG4 enhances integrin activation leading to the formation of a complex with signal transduction molecules such as focal adhesion kinase (FAK), which is a key factor for initial cell spreading [55]. Through a FAK-independent mechanism, CSPG4 could increase the activation of ERK 1 and 2 and sustaining cancer cell migration also through this pathway [52]. Activated CSPG4 can as well recruit the tyrosine-phosphorylated p130^cas^, an adaptor protein involved in the linkage of actin cytoskeleton to the extracellular matrix during cell migration, invasion and transformation [56, 57], contributing in this way to cytoskeletal reorganization and metastatic spread of CSPG4-positive tumor cells. In addition, several studies have demonstrated a role for CSPG4 in mediating multi-drug resistance via stimulation of PI3K/Akt signaling [58, 59]. Finally, it is thus not surprising that CSPG4 is overexpressed on CIC [46, 60] and tumor-derived exosomes [61] that are emerging as major players in cancer development and progression, contributing to recurrences, metastasis formation and chemoresistance [62–64].

## CSPG4 oncoantigen: a new star on the stage of comparative oncology

In recent years, the study of naturally occurring tumors in pet animals as models of human cancer, i.e. the comparative oncology, has been on the rise. Indeed, the difficulties in translating into the clinics several immunotherapeutic strategies found to be effective in rodents [6, 7] prompted the National Cancer Institute’s Center for Cancer Research [8] and, later on, a European Group [10] to launch the Comparative Oncology Program in order to find more informative pre-clinical models. In particular, the long history of dogs in biomedical research, their strong anatomical and physiological similarities to humans and the high number of pet dogs that are diagnosed with cancer and managed therapeutically, make these companion animals an attractive comparative model. Canine tumors mimic the progression of human malignancies better than any other pre-clinical model available so far since they grow over long periods of time following the natural evolution of human tumors, give rise to recurrences and metastases, and provide similar response to conventional therapies [9, 65, 66]. As a result, the study of spontaneous tumors developing in dogs as models for human malignancies is a priceless translational tool for accelerating the development of novel immunotherapeutic strategies with a substantial impact on the management of both canine and human oncological patients. Several canine tumors are “under the microscope” of comparative oncology for their translational relevance; among them there are lymphosarcomas, mammary carcinomas, melanomas and osteosarcomas [9, 67–70].

At the light of the comparative oncology concepts and considering the biological role of the CSPG4 oncoantigen in human tumors and its high conservation in structural and functional properties through phylogenetic evolution, the identification of CSPG4 in canine tumors could represent an unprecedented opportunity to pre-clinically test anti-CSPG4 immunotherapies in the veterinary field, highly predictive of their clinical efficacy in the human oncology. With this in mind and considering the well-known link between CSPG4 expression and melanoma progression in humans, other than the urgent need of effective strategies for the treatment of both human and canine melanomas [71–73], we were the first to evaluate CSPG4 expression in canine malignant melanomas [74]. About 60% of canine melanomas stained positive for CSPG4. The staining was mostly restricted to the tumor cell membrane with a different grade (or score) of expression in the different subtypes, being higher in epithelioid and in the more aggressive amelanotic phenotype. These findings labeled CSPG4 as a new potential marker for canine malignant melanoma diagnosis and as a promising candidate antigen for translational immunotherapy studies in dogs.

**Fig. 2:**
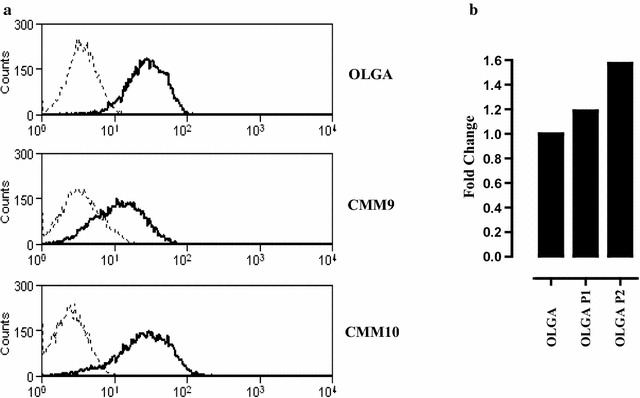
CSPG4 expression in canine melanoma cells and derived-CIC. **a** CSPG4 expression levels in three canine melanoma cell lines: OLGA [121], CMM9 and CMM10 (kind gift from Dr. Sasaki Nobuo and Dr. Nakagawa Takayuki, Laboratory of Veterinary Surgery, University of Tokyo, Japan). Cells were maintained in Dulbecco’s modified Eagle’s medium (DMEM; Sigma-Aldrich) supplemented with 10% fetal bovine serum (FBS; Sigma-Aldrich), 50 U/mL penicillin, and 50 μg/mL streptomycin (both from Invitrogen) in humidified incubator at 37 °C under 5% CO_2_. 2 × 10^5^ cells were incubated with a mix of CSPG4-specific mAb (225.28, VF4-TP108, VF20-TP108 and VF20-VT20; kindly provided by Prof. Soldano Ferrone, Massachusetts General Hospital, Boston, MA, USA) for 1 h at 4 °C. After washing with PBS, cells were incubated with a FITC-conjugated anti-mouse secondary antibodies for 30 min at 4 °C. Flow cytometry was performed with a CyAn ADP (DakoCytomation) and the results were analyzed with Summit 4.2 (DakoCytomation) software. *Black lines* show CSPG4 expression, while *dotted grey lines* show the background of cells stained with FITC-conjugated anti-mouse secondary antibody alone. A representative staining of three independent experiments is reported. **b** For CIC-enrichment, epithelial melanoma cells were detached by using non-enzymatic and mechanical dissociation and plated in ultra-low-attachment flasks at 6 × 10^4^ viable cells/mL in serum-free DMEM-F12 medium supplemented with 20 ng/mL basic fibroblast growth factor (FGF), 20 ng/mL epidermal growth factor (EGF), 5 µg/mL insulin, and 0.4% bovine serum albumin (BSA). Non-adherent spherical clusters of cells (P1), were collected after 7 days and disaggregated using non-enzymatic and mechanical dissociation. P1-derived single-cell suspensions were seeded again at 6 × 10^4^ viable cells/mL to generate non-adherent spherical clusters of cells (P2). 1 μg of RNA extracted from OLGA, P1-OLGA and P2-OLGA was retrotranscribed using RETROscript™ reagents (Ambion) and qPCR was carried out using gene-specific primers (Qiagen). Data were analyzed using SDS software 2.3 (Applied Biosystems). Relative CSPG4 gene expression was quantified using the threshold cycle (CT) value and normalized to housekeeping RNA18S. Relative expression of CSPG4 gene in the P1-OLGA and P2-OLGA compared with OLGA epithelial cells was calculated according to the method of Fold Change (2^−(DeltaDelta CT)^). Results representative of one out of three independent experiments is reported

Moreover, we have evaluated the presence of CSPG4 on different cell lines generated from three canine melanoma patients: one named OLGA, generated starting from the bioptic material obtained from a metastatic lymph node, and two lines named CMM9 and CMM10, derived from primary oral melanomas. CSPG4 expression on tumor cell membrane has been confirmed by flow cytometry analysis in all the canine melanoma cell lines analyzed (unpublished data, Fig. 2a), thus representing an interesting tool for the in vitro study of CSPG4 in a canine melanoma model. Besides, since in human tumors CSPG4 expression has been associated with the highly tumorigenic CIC subpopulation [46], we evaluated the expression of CSPG4 also on CIC-enriched melanospheres obtained from canine cell lines. Preliminary results suggest a CSPG4 over-expression at mRNA level in the first two in vitro passages of OLGA-derived melanospheres (P1 and P2) as compared to the epithelial counterpart (unpublished data, Fig. 2b), confirming CSPG4 as an attractive immunotherapeutic target potentially effective against recurrences and metastases in dogs.

**Fig. 3:**
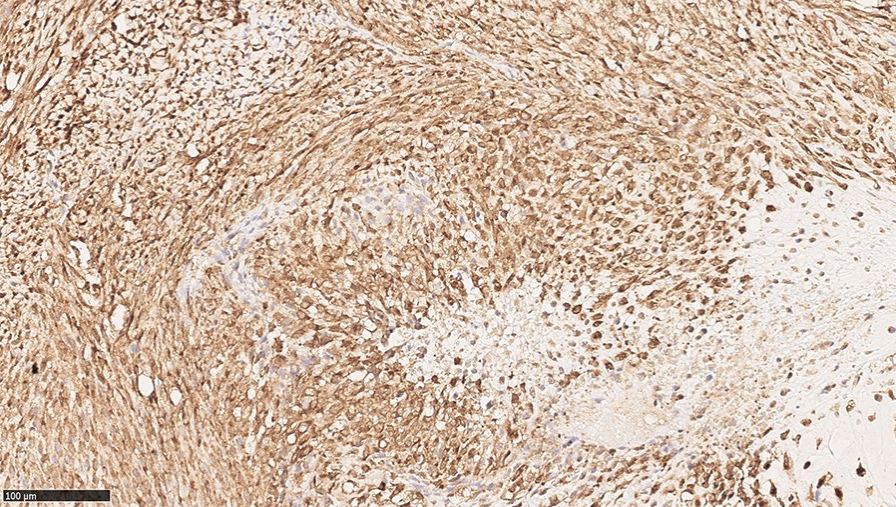
CSPG4 expression in canine osteosarcoma. Tissue samples from 29 canine osteosarcomas collected at the Diagnostic Laboratory of the Department of Animal Pathology of the University of Turin were examined. Data regarding breed, sex, age, tumor localization and clinical TNM staging were available for all dogs. The sample was fixed in 4% neutral buffered formalin, embedded in paraffin, and sectioned at 4 µm. Immunohistochemical analysis for CSPG4 was performed as previously described [74]. Briefly, sections were exposed to high-temperature antigen unmasking by incubation at 98 °C with citric acid buffer, pH 6.0. Endogenous peroxidase activity was blocked with 3% hydrogen peroxide in methanol for 30 min at room temperature. Tissue sections were incubated for 12 h at room temperature with a polyclonal anti-CSPG4 antibody (diluted 1:40, Sigma Aldrich), then 30 min with biotinylated-secondary antibody (Vectastain Elite ABC) and revealed with the ImmPACT DAB kit for peroxidase. A total score considering the proportion of positively stained tumor cells and the average staining intensity was assigned as previously described [74]. Briefly, the score indicating the positivity of tumor cells was assigned as follow: 0 (none); 1 (<1/100 or <1%); 2 (1/100–1/10 or 1–10%); 3 (1/10–1/3 or 10–30%); 4 (1/3–2/3 or 30–70%); and 5 (>2/3 or >70%). The score representing the estimated average staining intensity of positive tumor cells encompass *0* if none, *1* weak, *2* intermediate, *3* strong. The two scores were then added to each other to obtain a final score of CSPG4 expression ranging from 2 to 8. A representative image from a canine appendicular osteosarcoma is shown. Neoplastic cells are characterized by diffuse and strong cytoplasmic and membrane immunolabeling for CSPG4 and the total expression score is 8, resulted by the sum of the percentage of positive cells (=5) and the staining intensity (=3). Magnification 20X

Finally, as already shown for human tumors, it is likely that CSPG4 is expressed by other canine tumor histotypes besides malignant melanoma. Indeed, our immunohistochemical analysis of CSPG4 expression performed on a cohort of 29 canine osteosarcoma patients, revealed 23 (79%) positive and 6 (21%) negative cases. Particularly, the positivity score assigned as previously described [74] among the 23 CSPG4-expressing osteosarcomas was distributed as follow: 8 with a score of 8 (34.8%), 5 with a score of 7 (21.7%), 5 with a score of 6 (21.7%), 3 with a score of 5 (13.0%) and 2 with a score of 4 (8.7%). These preliminary data suggest a widespread expression of CSPG4 in appendicular osteosarcomas (unpublished data, Fig. 3), the most common primary canine malignant bone tumor [75].

All together, these findings highlight the great value of CSPG4 as a translational immunotherapeutic target in veterinary clinical practice, not only for melanoma but also for other tumor types.

## CSPG4 immune-targeting

The development of anti-cancer therapies directed against CSPG4 represents an unprecedented opportunity to simultaneously target tumor cells, CIC and pericytes on tumor vasculature. Moreover, since CSPG4 orchestrates multiple intracellular signaling pathways [35], its targeting could concurrently impair different oncogenic features of tumor cells. For these reasons several immunotherapeutic approaches against CSPG4 for the treatment of melanoma and other CSPG4-expressing tumor histoypes have been tested both in pre-clinical and clinical settings (Table 1).

**Table 1:** CSPG4 pre-clinical and clinical studies cited in the text

References	Cancer type	Methods/therapy	Study phase	Principal evidences
Burns et al. [86]	Melanoma	CAR-T cells generated from mAb 225.28S	Pre-clinical, in vitro	CAR-T cells are reactive against CSPG4-expressing cells and explanted human melanomas
Geldres et al. [85]	Melanoma, HNSCC, BC	CAR-T cells generated from mAb 763.74	Pre-clinical, in vitro and in vivo	CAR-T cells are cytotoxic against a variety of CSPG4-expressing cells and inhibit tumor growth
Beard et al. [87]	GB, mesothelioma, BC, osteosarcoma, melanoma, GB-derived CIC	CAR-T cells generated from mAb 225.28S, TP41.2, 149.53 and G71.1	Pre-clinical, in vitro	CAR-T cells demonstrate cytokine secretion and cytolytic function
Schmidt et al. [89]	Melanoma	CAR-T cells generated with 61scFv	Pre-clinical, in vitro and in vivo	CAR-T cells specific for CD20^+^CSPG4^+^ cells induce tumor eradication through targeted elimination
Erfurt et al. [91, 92]	Melanoma	CD4+ T cell isolated from healthy donors and patients	Pre-clinical, in vitro	Identification of CSPG4 peptide-specific CD4+ T cells reactive against melanoma cells
Rivera et al. [45]	Mesothelioma	mAb TP41.2	Pre-clinical, in vitro and in vivo	mAb treatment inhibits adhesion, motility, invasiveness of cancer cells and tumor growth
Wang et al. [46]	TNBC	mAb 225.28	Pre-clinical, in vitro and in vivo	mAb treatment inhibits adhesion and migration of cancer cells and tumor recurrences/metastasis
Poli et al. [95]	GB	Combinatorial treatment with mAb9.2.27 and NK cells	Pre-clinical, in vivo	Combination treatment inhibits tumor growth through immunological mechanisms
de Bruyn et al. [98]	Melanoma	Bifunctional fusion protein between mAb 9.2.27 and soluble human TRAIL	Pre-clinical, in vitro and in vivo	Bifunctional fusion protein induces the apoptosis of cancer cells and the inhibition of tumor growth
Bluemel et al. [99]	human-CSPG4 transected CHO cells	Different mAb for the generation of CSPG4/CD3-bispecific antibodies (BiTE)	Pre-clinical, in vitro	BiTE antibodies redirect the lysis of CSPG4^+^ cells according to the position of epitope binding domains
Torisu-Itakura et al. [100]	Melanoma	CSPG4/CD3-bispecific BiTE	Pre-clinical, in vitro	BiTe antibodies redirect the lysis of melanoma cells engaging patient-derived T cells
Amoury et al. [101]	TNBC	CSPG4-specific single-chain mAb 9.2.27 fragment fused to MAP tau	Pre-clinical, in vitro and in vivo	Fusion construct induces cytotoxic effects on TNBC cancer cells and the inhibition of tumor growth
Chekenya et al. [58]	GB	CSPG4 sh-induced KD	Pre-clinical, in vitro and in vivo	CSPG4 is associated with multi-drugs resistance and tumor growth through α3β1 integrin/PI3 K signaling
Yu et al. [102]	Melanoma	mAb 225.28	Pre-clinical, in vitro	mAb treatment enhances the in vitro efficacy of Braf-mediated inhibition of cancer cells
Mittelman et al. [104]	Melanoma	Vaccination with mouse anti-idiotypic mAb MF11-30	Clinical, in vivo	MF11-30 is safe, immunogenic and induces minor response in stage IV melanoma patients
Mittelman et al. [105]	Melanoma	Vaccination with mouse anti-idiotypic mAb MK2-23	Clinical, in vivo	MK2-23 is immunogenic and induces survival prolongation and metastasis regression
Wang et al. [106]	Melanoma	Vaccination with mouse anti-idiotypic mAb MK2-23 conjugated to IL-2	Pre-clinical, in vivo	IL-2 conjugation to MK2-23 is critical to induce an effective humoral and cellular response
Riemer et al. [107]	Melanoma	Vaccination with mAb 225.28-selected mimotope fused with streptococcal ABP	Pre-clinical, in vitro and in vivo	Mimotope is immunogenic and reactive against CSPG4^+^ melanoma cells
Wagner et al. [108]	Melanoma	Vaccination with mAb 225.28-selected mimotope fused with tetanus toxoid	Pre-clinical, in vitro and in vivo	Mimotope is immunogenic and reactive against CSPG4^+^ melanoma cells
Luo et al. [109]	Melanoma	Vaccination with peptide P763.74 mimicking CSPG4	Pre-clinical, in vitro and in vivo	P763.74 inhibits melanoma cells migration through immunological and non-immunological mechanisms
Piras et al.[96], Riccardo et al. [9]	Melanoma	DNA electrovaccination	Pre-clinical, in vitro and in vivo	Anti-CSPG4 DNA vaccination is immunogenic and clinically effective in canine melanoma patients

*HNSCC* head and neck squamous-cell carcinoma, *BC* breast cancer, *GB* glioblastoma, *TNBC* triple negative breast cancer, *KD* knock-down

One explored strategy in the immunotherapy field, consisting in the stimulation of autologous tumor-specific lymphocytes ex vivo to improve cell mediated immunity before adoptively transferring them back to the patient, were first described almost four decades ago. Since then, several attempts to treat metastatic melanoma were carried out exploiting the adoptive cell transfer (ACT) of autologous lymphocytes derived from the tumor or from the blood of patients [76–80]. Despite the induction of an objective cancer regression in a measurable proportion of treated patients, this strategy has several limitations, including the requirement of pre-existing antitumor-reactive cells that should be expanded ex vivo and the inadequate applicability in other cancer histotypes other than melanoma. The possibility of genetically engineering T cells with conventional T-cell receptors (TCRs) or chimeric antigen receptors (CARs) and consequently redirecting T lymphocytes to recognize and destroy specific TAA has opened up new opportunities for the utilization of ACT to treat different types of cancer patients [81]. Indeed, these T cell-based approaches have been already used in pre-clinical and clinical trials for the treatment of several types of malignancies, including melanoma, myeloma and haematologic malignancies [82–85]. On the wave of these novel approaches, CSPG4 has been considered a suitable target for CAR-T cells. Indeed, several CSPG4-specific CARs have been generated by utilizing monoclonal antibodies (mAbs) reactive against CSPG4 and pre-clinically demonstrated anti-tumor activity against not only melanoma, but also against many other CSPG4-positive cancer histotypes, including breast carcinoma, head and neck squamous cell carcinoma and mesothelioma, as well as against CIC [85–88]. Moreover, Smidth and collaborators interestingly changed the prospective of ACT therapy, demonstrating that the adoptive transfer of CARs directed against CD20 and CSPG4 molecules—co-expressed by less that 2% of melanoma cells—is effective in the eradication of tumor lesions, while the targeting of any other minor subset is less effective [89]. In this way they revealed that melanoma lesions can be efficiently eradicated through the specific T cell-mediated elimination of a definite melanoma cell population, highlighting the fundamental relevance of CSPG4 targeting. Indeed, redirecting T cells to CSPG4 using CARs may represent a robust immunotherapeutic approach to target multiple solid tumors; however, till now, the clinical experience with engineered T cells is limited and some challenges have still to be overcome [90].

The importance of CSPG4 as a promising target for T cell-based immunotherapy has been also supported by Erfurt and colleagues, which demonstrated the presence of a CD4^+^ T cells reactivity against specific peptides located in the extracellular domain of CSPG4 antigen in the peripheral blood of both healthy donors and melanoma patients, in the absence of clinical signs of autoimmunity. Importantly, these peptide-specific CD4^+^ T cells could strongly recognize CSPG4 expressing melanoma cells, suggesting that the identified peptides are naturally processed by tumor cells. These findings supported the idea that the activation and the expansion of CSPG4-reactive CD4^+^ T cells circulating in the blood could be considered for further development of anti-CSPG4 immunotherapeutic strategies [91, 92].

However, since in several malignancies tumor cells have been reported to escape T-cell recognition [93, 94], more and more researchers have focused their attentions on mAb-based therapies and several studies have investigated the potentiality of mAb-based anti-tumor strategies directed against CSPG4. This body of findings solidly demonstrated, in different cancer cells and in various experimental settings, the efficacy of anti-CSPG4 mAb in impairing cancer cell proliferation, migration and invasion. These effects are mediated by multiple mechanisms, including direct mAb activity, such as the induction of CSPG4 down-regulation and a reduced activation of CSPG4-dependent signaling pathways, and mAb dependent recruitment and activation of immune-effector mechanisms [45, 60, 95, 96]. More sophisticated mAb-based approaches, including chimeric anti-CSPG4 antibodies fused to super-antigens, bi- and tri-specific antibodies, or cytolytic fusion protein of CSPG4-specific single-chain antibody fragment genetically fused to microtubule-associated protein (MAP) tau, were also exploited [97–101], confirming the efficacy of the immune-targeting of CSPG4-positive cancer cells. Recent studies have also provided evidences of the positive impact of anti-CSPG4 mAb in improving the efficacy of anti-cancer drugs in glioblastoma and melanoma pre-clinical models, indirectly associating the CSPG4 expression with multidrug resistance in these tumor histotypes [58, 102, 103].

An alternative approach to mAb administration is obviously active immunization, which has the potential to bring about effective and long-lasting anti-tumor responses without significant side effects and the risk of resistance development. First evidences of the effectiveness of active immunization against CSPG4 in melanoma patients were achieved through vaccination with the anti-idiotypic antibody MK2-23, which bears the internal image of the mAb 763.74 against a defined CSPG4 epitope. Interestingly, the induction of CSPG4-specific antibodies in immunized patients was associated with significantly longer survival and metastasis regression [104, 105]. However, this approach never ended up in clinics, due to both the difficulties in standardization of MK2-23 (it has to be conjugated to keyhole limpet hemocyanin as a carrier) and to side effects associated with Bacille Calmette–Guerin administration required to induce an efficient immune response [106]. Alternative strategies evaluating in a pre-clinical setting the impact of the fusion of mAb MK2-23 with IL-2 demonstrated an enhanced immunogenicity of the novel construct and thus the possibility to bypass the requirement for conjugation to a carrier and administration with an adjuvant [106]. Moreover, anti-CSPG4 vaccination was again of interest when the mimotope technology emerged, indeed immunizations with CSPG4 mimotopes resulted in an inhibition of proliferation, migration and invasion of CSPG4-positive melanoma cells through the induction of a specific antibody response responsible for both immunological and non-immunological antitumor functions [107–109].

Overall, these are encouraging data providing a strong rationale for the development of new strategies of active immunization against CSPG4.

## DNA vaccination against CSPG4: a strategy for the treatment of canine malignant melanoma with translational implications

Amongst the various possible approaches for active immunization, DNA vaccination has some advantages over other strategies. Among them there are the high stability of DNA and the possibility to generate the vaccine in large amounts in a cheaper and faster manner than other vaccines [110]. DNA vaccines consist in circular DNA constructs (plasmids) that encode one or more TAA. Thanks to its bacterial derivation and to the presence of hypomethylated CpG dinucleotide-containing motifs, the plasmid is able per se to stimulate the innate immune system by interacting with toll-like receptor (TLR)-9 [111] augmenting the antigen-specific immune response. The stimulation of a range of TLR9-expressing cells, including dendritic cells (DC) and B cells, can create an inflammatory milieu for triggering the adaptive immune response [112]. Plasmids administration intradermally, subcutaneously or intramuscularly, results in transfection of resident cells, including DC and other antigen-presenting cells. This allows TAA expression and presentation on both MHC Class I and Class II molecules and stimulation of the cellular and the humoral arms of the immune system against TAA-positive cancer cells (reviewed in [113]).

A large body of studies assessed the efficacy and the safety of the DNA vaccination technique for cancer treatment and prevention in a variety of animal models and even in humans with plasmids encoding different TAA [114–120]. However, overall clinical benefit has been so far limited, despite the high efficacy of DNA vaccines in pre-clinical models [110]. A critical issue linked to the high failure of DNA vaccination approaches in the clinical trials could be related to the way of vaccine administration and design. The intramuscular administration of the DNA vaccine in association with electroporation is one of the most effective way of immunization identified so far [114]. Another major challenge in the development of successful cancer DNA vaccines lies in the fact that most oncoantigens, including CSPG4, are non-mutated and tolerated self-proteins. Therefore, there is the need of identifying a strategy to overcome the immune-tolerance normally existing in patients, in order to induce a proper long-term immune response towards self-antigens. The use of DNA plasmids coding for xenogeneic proteins is one interesting strategy widely investigated in several pre-clinical and clinical studies that have demonstrated its efficacy [121–124]. Moreover, xenovaccination has recently been shown to improve survival in veterinary cancer patients, mainly in dogs affected by spontaneous disease [96, 121, 125, 126]. Positive results obtained in veterinary trials led to the approval by the US FDA of the first xenogeneic DNA vaccine against tyrosinase, ONCEPT (Merial), for the treatment of oral malignant melanomas in dogs [5]. Although the therapeutic efficacy of ONCEPT has been recently questioned [127], its licensing has elicited enthusiasm for this immunization strategy as a potentially effective immunotherapeutic approach. Indeed, the use of a xenogeneic antigen could circumvent the immune tolerance because it preserves a high degree of similarity and, at the same time, is different enough from the targeted self-oncoantigen. Those differences between epitopes of the orthologue and the native protein are responsible for eliciting T and B cell responses against the xenoantigen that may cross-react with the self-target. This makes the DNA vaccine highly immunogenic and consequently more likely effective.

The ability of xenogeneic DNA vaccines to break the immune tolerance and to induce an effective immune response against a self-oncoantigen has been extensively demonstrated also by us [96, 114, 124, 128] and was applied to our anti-CSPG4 vaccine. Indeed, we have recently demonstrated the immunogenicity, safety and therapeutic efficacy of the electroporation of a xenogeneic DNA vaccine coding for the human CSPG4 (Hu-CSPG4) protein in prospectively enrolled client-owned dogs with *en bloc* surgically resected stage II and III CSPG4-positive spontaneous oral malignant melanoma [96, 121]. The choice to use the human sequence of CSPG4 was due to the high conservation of CSPG4 through evolution, with 82% of homology and 88% of similarity of the Hu-CSPG4 to the canine counterpart (Do-CSPG4). The results obtained in our studies demonstrate the ability of the xenogeneic DNA vaccine against CSPG4 to induce a specific humoral response against the human protein coded by the plasmid and against the canine counterpart. This antibody response relates favorably with the significant prolongation of disease-free and overall survival time in vaccinated dogs with surgically resected malignant melanoma as compared to controls treated with surgery alone [96, 121].

## Conclusions

Our results indicate that CSPG4 xenogeneic DNA vaccination associated to electroporation in dogs affected by MM is safe and able to overcome host unresponsiveness to the self-antigen, resulting in significantly increased overall and disease-free survival, thanks to the induction of anti-CSPG4 antibodies. These findings corroborate the clinical impact of CSPG4 immune-targeting for the management of canine melanoma patients. Moreover, the recognition of CSPG4 expression in other aggressive and incurable tumor histotypes may promote the spread of anti-CSPG4 vaccination strategy for the treatment of the wider population of CSPG4-positive tumor affected dogs. Finally, the recognized value of spontaneous tumors in dogs as a priceless model for predicting tumor behavior and response to immunotherapy in humans, highlights the translational potential of active CSPG4 immune-targeting that might be revolutionary for the management of both canine and human CSPG4-positive cancer patients.

## Abbreviations

TAA: tumor-associated antigens
FDA: Food and Drug Administration
CSPG4: chondroitin sulfate proteoglycan-4
CIC: cancer initiating cells
HMW-MAA: high molecular weight-melanoma associated antigen
MCSP: melanoma chondroitin sulfate proteoglycan
NG2: nerve/glial antigen 2
bp: base pair
aa: amino acids
CS: chondroitin sulfate
ECM: extracellular matrix
LGR: laminin G-type regions
FGF: fibroblast growth factors
PDGF: platelet-derived growth factor
ERK: extracellular signal-regulated kinase
PRR: proline-rich region
RTK: tyrosine kinase receptors
MAPK: mitogen-activated protein kinases
FAK: focal adhesion kinase
ACT: adoptive cell transfer
TCRs: T cell receptors
CARs: chimeric antigen receptors
mAb: monoclonal antibody
MAP: microtubule-associated protein
TLR: toll-like receptor
DC: dendritic cells
DMEM: Dulbecco’s modified Eagle’s medium
FBS: fetal bovine serum
BSA: bovine serum albumin
